# Efficacy of early vs. late use of frovatriptan combined with dexketoprofen vs. frovatriptan alone in the acute treatment of migraine attacks with or without aura

**DOI:** 10.1007/s10072-014-1751-3

**Published:** 2014-05-28

**Authors:** Gianni Allais, Vincenzo Tullo, Pietro Cortelli, Piero Barbanti, Fabio Valguarnera, Giuliano Sette, Florindo D’Onofrio, Marcella Curone, Dario Zava, Deborha Pezzola, Giorgio Reggiardo, Stefano Omboni, Fabio Frediani, Gennaro Bussone, Chiara Benedetto

**Affiliations:** 1 Women’s Headache Center, Department of Surgical Sciences, University of Turin, Via Ventimiglia 3, 10126 Turin, Italy; 2 Clinical Neuroscience, National Neurological Institute Carlo Besta, Milan, Italy; 3 Neurological Science, University of Bologna, Bologna, Italy; 4 IRCCS San Raffaele Pisana, Rome, Italy; 5 Sestri Ponente Hospital, Genoa, Italy; 6 Sant’Andrea Hospital, Rome, Italy; 7 Neurologic Unit, San Giuseppe Moscati Hospital, Avellino, Italy; 8 Istituto Lusofarmaco d’Italia, Medical Department, Milan, Italy; 9 Mediservice, Milan, Italy; 10 Clinical Research Unit, Italian Institute of Telemedicine, Varese, Italy; 11 Ospedale San Carlo Borromeo, Milan, Italy

**Keywords:** Migraine, Frovatriptan, Dexketoprofen, Early intake, Late intake

## Abstract

Early triptan use after headache onset may help improve the efficacy of acute migraine treatment. This may be particularly the case when triptan therapy is combined with a nonsteroidal anti-inflammatory drug (NSAID). The objective of this is to assess whether the combination of frovatriptan 2.5 mg + dexketoprofen 25 or 37.5 mg (FroDex25 and FroDex37.5) is superior to frovatriptan 2.5 mg alone (Frova) in the acute treatment of migraine attacks in patients who took the drug within 30 min from the onset of pain (early use) or after (late use). A total of 314 subjects with a history of migraine with or without aura were randomized into a double-blind, multicenter, parallel group, pilot study to Frova, FroDex25 or FroDex37.5 and were required to treat at least one migraine attack. In the present post hoc analysis, traditional migraine endpoints were compared across study drugs for subgroups of the 279 patients of the full analysis set according to early (*n* = 172) or late (*n* = 107) drug use. The proportion of patients pain free at 2 h in the early drug use subgroup was 33 % with Frova, 50 % with FroDex25 and 51 % with FroDex37.5 mg (*p* = NS combinations vs. monotherapy), while in the late drug use subgroup was 22, 51 and 50 % (*p* < 0.05 FroDex25 and FroDex37.5 vs. Frova), respectively. Pain-free episodes at 4 h were 54 % for early and 34 % for late use of Frova, 71 and 57 % with FroDex25 and 74 and 68 % with FroDex37.5 (*p* < 0.05 for early and *p* < 0.01 for late use vs. Frova). The proportion of sustained pain free at 24 h was 26 % under Frova, 43 % under FroDex25 mg and 40 % under FroDex37.5 mg (*p* = NS FroDex25 or 37.5 vs. Frova) in the early drug intake subgroup, while it was 19 % under Frova, 43 % under FroDex25 mg and 45 % under FroDex37.5 mg (*p* < 0.05 FroDex25 and FroDex37.5 vs. Frova) in the late drug intake subgroup. Risk of relapse at 48 h was similar (*p* = NS) among study drug groups (Frova: 25 %, FroDex25: 21 %, and FroDex37.5: 37 %) for the early as well as for the late drug use subgroup (14, 42 and 32 %). FroDex was found to be more effective than Frova taken either early or late. The intrinsic pharmacokinetic properties of the two single drug components made FroDex combination particularly effective within the 2–48-h window from the onset of the acute migraine attack. The efficacy does not seem to be influenced by the time of drug use relative to the onset of headache.

## Introduction

Early triptan use after the onset of headache may help to improve the efficacy of acute migraine treatment, particularly in those patients with rapid pain onset and worsening, high frequency of pain recurrence and severe associated symptoms [1–3]. Despite their utility as migraine abortive medications, however, the triptans do not successfully treat all attacks of migraine or relieve all migraine associated symptoms, even when they are administered in the early phase of the acute attack [4].

A possible solution to increase the chance of successful treatment is to combine the triptan with a nonsteroidal anti-inflammatory drug (NSAID), which may help to effectively target the distinct vascular and inflammatory processes underlying migraine [4, 5]. Studies combining sumatriptan with naproxen [6–8], rizatriptan with rofecoxib [9] or almotriptan with aceclofenac [10] have all demonstrated an increase in the proportion of migraine patients with desirable treatment outcomes.

Recently, a randomized, double-blind, parallel group study documented an improved initial efficacy, but similar sustained pain free, when treating the acute attack with a combination of frovatriptan and dexketoprofen rather than with frovatriptan alone [11]. These results were most likely to be linked to the intrinsic pharmacokinetic properties of the two drugs: dexketoprofen is absorbed rapidly and contributes to the early efficacy of the combination whereas frovatriptan persists longer and so provides sustained efficacy with less recurrence [11–14].

In the present retrospective analysis of the aforementioned randomized, prospective study we made an initial determination of whether differences in the efficacy of the combination of frovatriptan with dexketoprofen over frovatriptan alone may exist with early or late use of the drugs (i.e. within or after 30 min from the onset of headache pain).

## Methods

### Study population and design

Full details of the study methodology are available in the original publication [11]. Briefly, the study enrolled male and non-pregnant and non-breast feeding female subjects, aged 18–65 years, with a current history of migraine with or without aura [15], and with at least one, but no more than six, migraine attacks per month for 6 months prior to entering the study. In the present retrospective analysis we separately selected subjects who treated headache pain within 30 min of its earliest onset, or when headache pain was established (late use, >30 min). This was a multicenter, randomized, double-blind, active-controlled, three parallel group, study, conducted in 25 different Italian Headache Centers. Following a screening visit eligible patients were randomized to frovatriptan 2.5 mg (Frova), or to extemporaneous combinations of frovatriptan 2.5 mg + dexketoprofen 25 mg (FroDex25) or frovatriptan 2.5 mg + dexketoprofen 37.5 mg (FroDex37.5). To ensure blinding the study drugs were overencapsulated. At the end of the randomization visit a headache diary was dispensed to the patient in order to document the characteristics of the headache pain and associated symptoms. The intensity of headache and the associated symptoms was graded according to a four-point rating scale, as recommended by International Headache Society [15]. Each subject was also given the study medication and was instructed to self-administer the drug at home and complete the diary, for the first migraine attack occurring during the study period (i.e. within 1 month from randomization).

### Data analysis

As in the original publication, this post hoc analysis was based on the full analysis set, including all subjects randomized and treated, for whom at least one post-dose headache attack was recorded. As aforementioned, the analysis was separately performed in the subgroup of patients reporting early or late study drug use.

The following efficacy endpoints were evaluated for each of the subgroups: (a) proportion of pain-free subjects at 2 h before any rescue medication (original primary study endpoint, estimated according to IHS Guidelines) [15]; (b) proportion of pain-free subjects at 4 h before any rescue medication [15]; (c) sustained pain free within 24 h (episode pain free at 2 h with no use of rescue medication or recurrence within 24 h); (d) relapse within 48 h (episode pain free at 2 h and headache of any severity returning within 48 h in a subject who did not take any rescue medication) [15]; (e) proportion of subjects taking rescue medication; and (f) subjects’ preference for treatment.

Continuous variables were summarized by computing average values and standard deviations (SD), while categorical variables by computing the absolute value and the frequency (as percentage). The primary study endpoint was assessed by the Fisher–Freeman–Halton Exact test statistics using either a 3 × 2 contingency table for testing association or a 2 × 2 contingency table for comparisons between treatments. The Fisher Exact test based on 2 × 2 contingency tables was applied also to secondary variables to check difference between pairs of treatments. A *t* test of Student was used to evaluate differences between continuous variables. All tests were two sided and the level of statistical significance was set at 0.05 for all analyses.

## Results

**Fig. 1:**
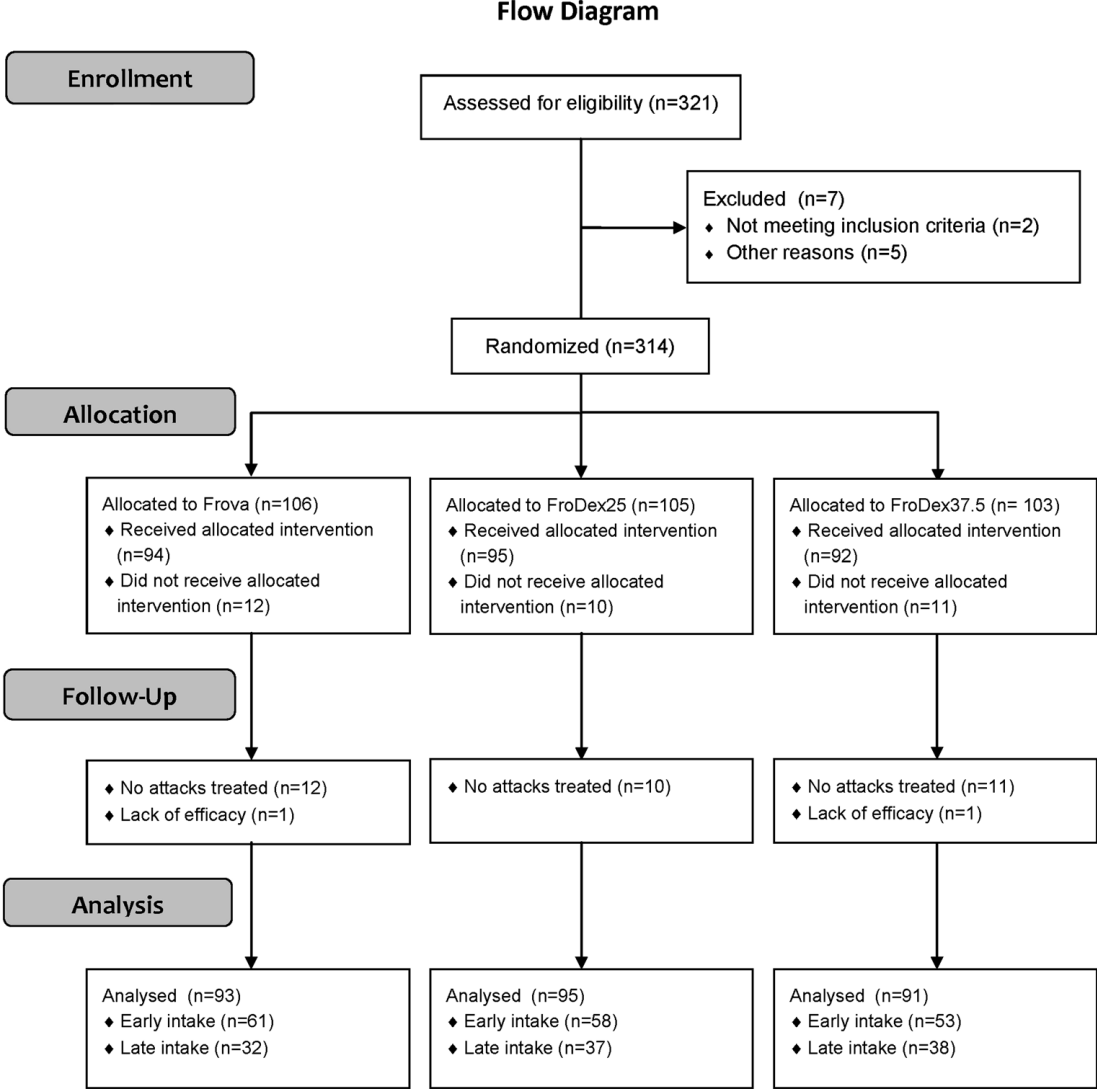
Flow diagram of the patients throughout the study

The flow diagram of the patients through the study is shown in Fig. 1. Of the 279 subjects of the full analysis set, 172 reported an early drug intake (61 Frova, 58 FroDex25 and 53 FroDex37.5) and 107 a late drug intake (32 Frova, 37 FroDex25 and 38 FroDex37.5). Table 1 shows the main demographic and clinical characteristics of the patients at randomization, by subgroups of patients according to time of first drug use and allocated treatment. No statistically significant difference was observed in any demographic or clinical characteristic, across the three study treatments for both the early and the late drug use subgroups. However, subjects in the early drug use subgroup had a significantly (*p* < 0.001) higher proportion of migraine attacks of severe intensity (39.0 vs. 16.8 % late drug intake), while those in the late drug intake group reported a higher rate of moderate intensity attacks (81.3 vs. 52.3 % early drug intake). Among the associated symptoms, phonophobia more frequently (*p* < 0.05) occurred in subjects in the early drug use group (66.9 vs. 54.2 %). Subjects with early drug use also reported a significantly (*p* < 0.01) lesser use of triptans prior to enrolment into the study (19.8 vs. 34.6 % late drug use).

**Table 1:** Demographic and clinical characteristics of the 279 patients of the full analysis set at the time of randomization

	Early drug use (≤30 min)				Late drug use (>30 min)			
	Frova (*n* = 61)	FroDex25 (*n* = 58)	FroDex37.5 (*n* = 53)	All (*n* = 172)	Frova (*n* = 32)	FroDex25 (*n* = 37)	FroDex37.5 (*n* = 38)	All (*n* = 107)
Age (years, mean ± SD)	38.5 ± 9.4	37.7 ± 10.7	40.0 ± 9.0	38.7 ± 9.7	39.6 ± 8.0	40.5 ± 9.5	41.6 ± 10.9	40.6 ± 9.6
Females (*n*, %)	58 (95.1)	52 (89.7)	44 (83.0)	154 (89.5)	30 (93.8)	32 (86.5)	30 (78.9)	92 (86.0)
Height (cm, mean ± SD)	164.9 ± 5.8	166.5 ± 6.9	166.7 ± 6.9	166.0 ± 6.5	163.3 ± 5.6	164.8 ± 8.5	165.8 ± 9.0	164.7 ± 7.9
Weight (kg, mean ± SD)	61.3 ± 8.3	61.8 ± 6.9	63.0 ± 11.2	62.0 ± 9.6	60.6 ± 9.6	60.9 ± 11.1	64.2 ± 13.6	62.0 ± 11.6
MIDAS score (mean ± SD)	25.6 ± 18.0	26.3 ± 10.7	24.1 ± 19.1	25.4 ± 23.7	18.2 ± 14.0*	24.6 ± 25.4	21.3 ± 12.5	21.5 ± 18.4
Presence of aura (*n*, %)	6 (9.8)	1 (1.7)	1 (1.9)	8 (4.7)	3 (9.4)	1 (2.7)	4 (10.5)	8 (7.5)
Intensity of baseline attack (*n*, %)								
Mild	8 (13.1)	5 (8.6)	2 (3.8)	15 (8.7)	–	1 (2.7)	1 (2.6)	2 (1.9)***
Moderate	32 (52.5)	30 (51.7)	28 (52.8)	90 (52.3)	26 (81.3)*	32 (86.5)**	29 (76.3)	87 (81.3)***
Severe	21 (34.4)	23 (39.7)	23 (43.4)	67 (39.0)	6 (18.8)*	4 (10.8)**	8 (21.1)	18 (16.8)***
Presence of nausea (*n*, %)	31 (50.8)	32 (55.2)	25 (47.2)	88 (51.2)	14 (43.8)	15 (40.5)**	17 (44.7)	46 (43.0)
Presence of photophobia (*n*, %)	42 (68.9)	41 (70.7)	39 (73.6)	122 (70.9)	22 (68.8)	20 (54.1)	24 (63.2)	66 (61.7)
Presence of phonophobia (*n*, %)	42 (68.9)	37 (63.8)	36 (67.9)	115 (66.9)	16 (50.0)	25 (67.6)	17 (44.7)*	58 (54.2)*
Preventive therapy (*n*, %)								
Antidepressant	4 (6.6)	2 (3.4)	6 (11.3)	12 (7.0)	5 (15.6)	6 (16.2)	4 (10.5)	15 (14.0)
Antiepileptics	5 (8.2)	5 (8.6)	5 (9.4)	15 (8.7)	2 (6.3)	1 (2.7)	5 (13.2)	8 (7.5)
Beta-blocking agents	3 (4.9)	1 (1.7)	4 (7.5)	8 (4.7)	1 (3.1)	2 (5.4)	5 (13.2)	8 (7.5)
Triptan users (*n*, %)	14 (23.0)	10 (17.2)	10 (18.9)	34 (19.8)	9 (28.1)	14 (37.8)*	14 (36.8)	37 (34.6)**
NSAIDs users (*n*, %)	17 (27.9)	9 (15.5)	8 (15.1)	34 (19.8)	8 (25.0)	6 (16.2)	7 (18.4)	21 (19.6)

Data are separately shown for the early (≤30 min) and late (>30 min) drug intake and by type of treatment, and are summarized as mean (±SD), or absolute (*n*) and relative frequency (%). *Asterisks* refer to the statistical significance of the difference between the early vs. late subgroup (* p < 0.05, ** *p* < 0.01 and *** *p* < 0.001)

*Frova* frovatritpan, *FroDex* frovatriptan + dexketoprofen, *MIDAS* migraine disability assessment, *NSAID* nonsteroidal anti-inflammatory drugs

**Fig. 2:**
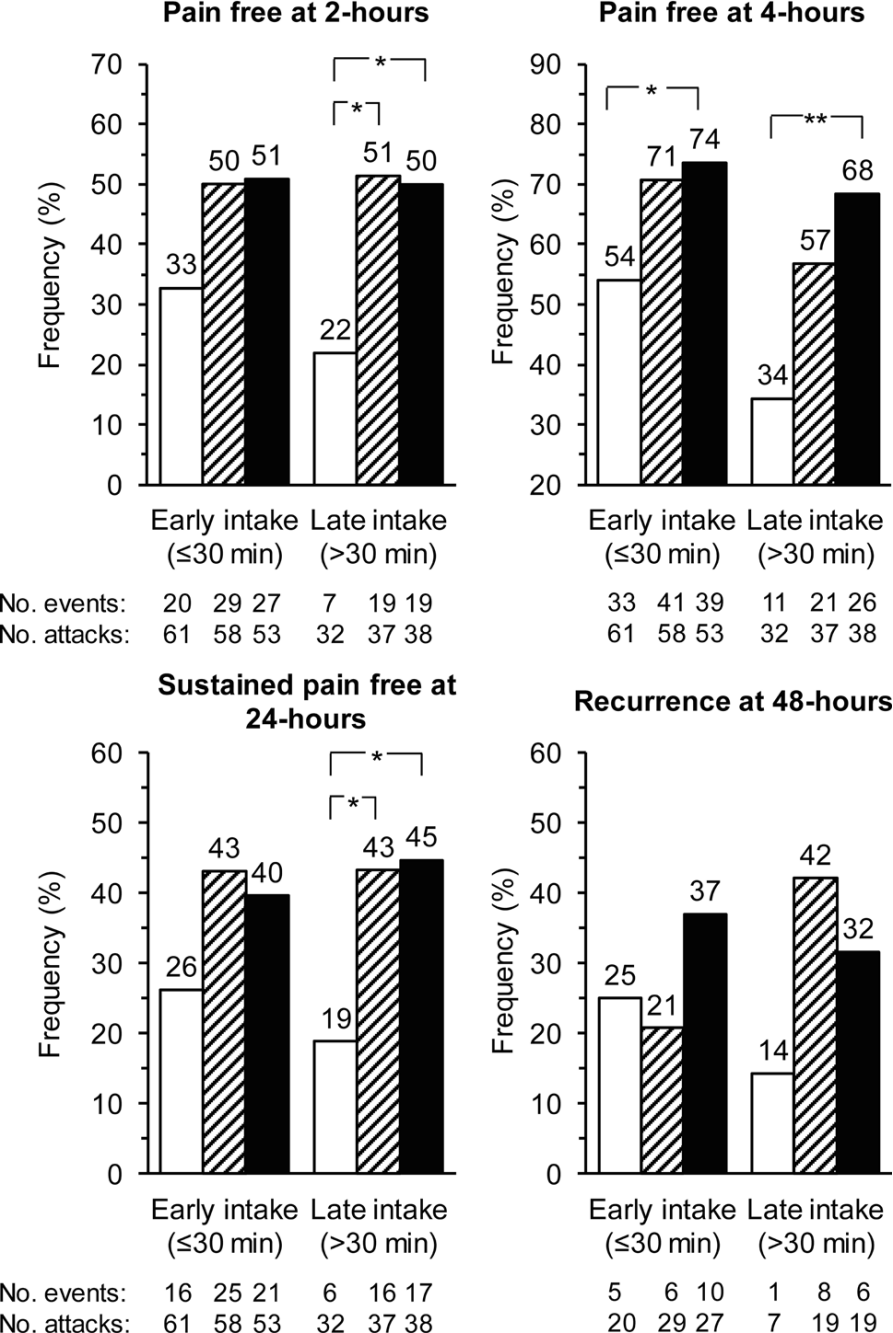
Proportion (%) of pain free at 2-h and at 4-h, sustained pain free at 24-h and recurrence at 48-h, after administration of frovatriptan 2.5 mg (*open bars*), frovatriptan 2.5 mg + dexketoprofen 25 mg (*striped bars*) and frovatriptan 2.5 mg + dexketoprofen 37.5 mg (*full bars*), separately shown for the patients reporting an early or a late drug intake. *Asterisks* indicate a statistically significant difference (*p < 0.05 and **p < 0.01) between the combination treatment and the monotherapy

Overall comparison among treatments for pain free at 2 h showed a statistically significant difference in favor of the combination therapy vs. the monotherapy for the late (*p* < 0.05, Fisher–Freeman–Halton Exact test on 3 × 2 contingency table), but not for the early drug use subgroup (Fig. 2). When pairs of treatments were compared, a statistically significant difference was observed in the late drug dosing subgroup between FroDex 25 and Frova (*p* < 0.05) and between FroDex37.5 and Frova (*p* < 0.05) (Fig. 2). In both study subgroups the proportion of pain free at 4 h was significantly better with FroDex37.5 than with Frova (*p* < 0.05 for the early and *p* < 0.01 for the late use subgroups, respectively) (Fig. 2). The proportion of sustained pain free within the 24 h was significantly (*p* < 0.05) larger under FroDex25 and FroDex37.5 than under the monotherapy in the late drug intake subgroup (Fig. 2). Finally, the proportion of recurrence within 48 h was similar between Frova and the combination therapy, either for the early or for the late intake subgroup (Fig. 2).

For pain free at 2 and 4 h, sustained pain free at 24 h and recurrence at 48 h, no statistically significant difference was ever observed between early and late drug users, although a trend was observed for a better efficacy in case of early intake for monotherapy-treated patients.

Recourse to rescue medication was not significantly different among the three treatment groups for patients with early drug intake (Frova: 25 of 61 patients, 41.0 %, FroDex25: 14/58, 24.1 % and FroDex37.5: 15/53, 28.3 %), while among those with late drug intake it was significantly (*p* < 0.05) lower with FroDex37.5 (11/38, 29.0 % vs. 17/32, 53.1 % Frova and 17/37, 46.0 % FroDex25).

**Fig. 3:**
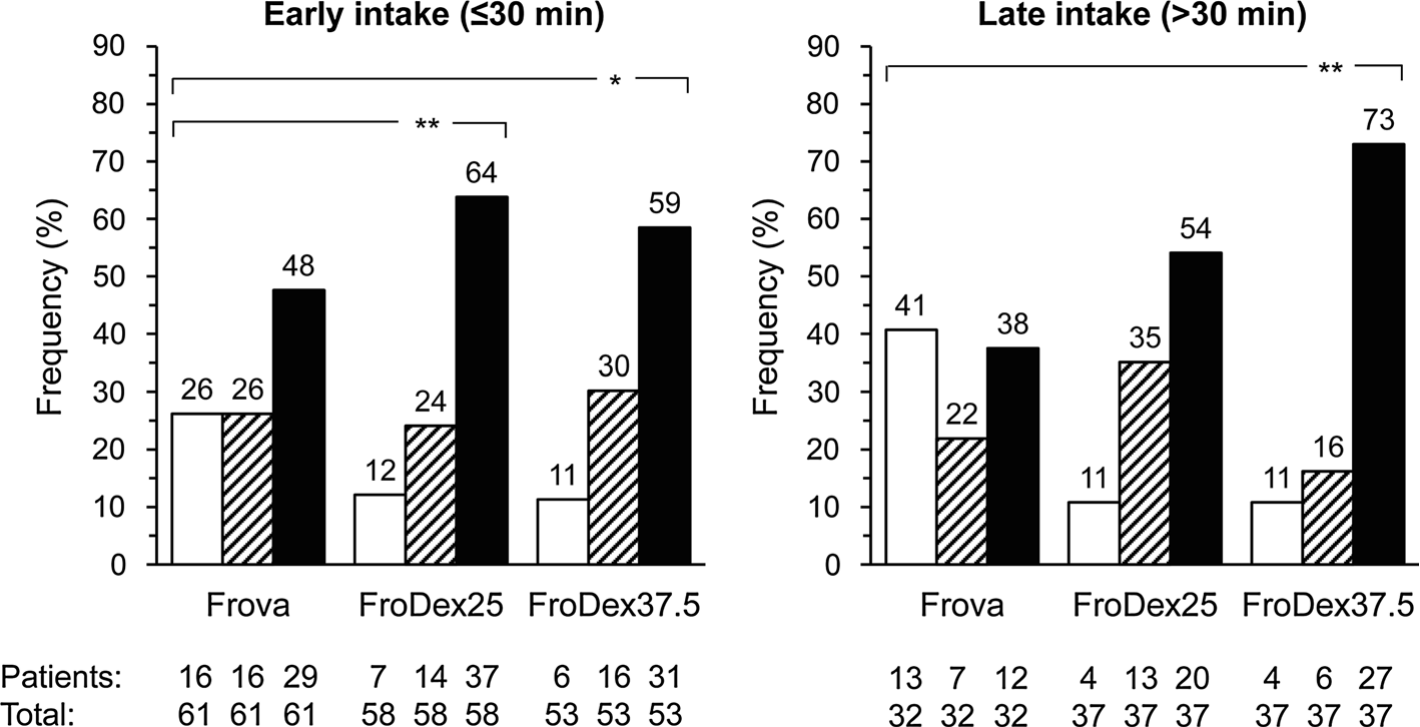
Proportion (%) of patients judging treatment poor or very poor (*open bars*), reasonable (*striped bars*) or good or excellent (*full bars*). Data are separately shown for the subgroup of patients reporting an early drug intake and for those with a late drug intake, and for the three different treatments (Frova: frovatriptan 2.5 mg; FroDex25: frovatriptan 2.5 mg + dexketoprofen 25 mg; FroDex37.5: frovatriptan 2.5 mg + dexketoprofen 37.5 mg). *Asterisks* indicate a statistically significant difference (*p < 0.05 and **p < 0.01) between the combination treatment and the monotherapy

Finally, treatment was judged excellent or good by significantly more patients under the combination treatment with respect to the monotherapy in the early intake (FroDex25: 37 of 58 patients, 63.8 % and FroDex37.5: 31/53, 58.5 % vs. Frova: 29/61, 47.6, *p* < 0.01 and *p* < 0.05, respectively) and in the late intake group (FroDex37.5: 27 of 37 patients, 73.0 % vs. Frova: 12/32, 37.5 %, *p* < 0.01) (Fig. 3).

## Discussion

In our post hoc analysis of a randomized, double-blind, active-controlled, dose comparison study [11], administration of the combination of frovatriptan 2.5 mg + dexketoprofen 25 or 37.5 mg showed a better efficacy than frovatriptan alone on the primary study end-point, pain free at 2 h, in the late drug, but not in the early drug users. Also the proportion of pain free episodes at 4 h was larger with the FroDex37.5 combination than with the monotherapy, in this case for both early and late drug users. In FroDex37.5-treated patients, the use of rescue medication was significantly lower than in monotherapy-recipients when the drug was used to treat the attack at a later stage. Sustained pain free within the 24 h was better in the combination treatment group, but yet only for patients in the late drug intake group. The proportion of relapse up to 48 h was similar in the three treatments arms and no differences were observed in early and late drug “dosers”.

These results taken together suggest that, when dexketoprofen is used in combination with frovatriptan, early or late intake does not affect response to treatment, whereas this is not the case when frovatriptan is used alone. Such a finding supports current recommendations advising administration of a triptan monotherapy as early as possible at the time of headache onset in order to ensure the best effect [16–18]. It also adds an important piece of evidence on the effectiveness of a combination between a triptan and NSAID, also when taken later after the onset of pain. In the case of the combination used in the present study, it is likely that the short half-life of dexketoprofen and its rapid onset of action may contribute to the high pain free response, whereas the sustained effect of the combination may be largely driven by the long-half life of frovatriptan [13, 14]. We may hypothesize that using a combination of a drug with a fast action (dexketoprofen) and of a drug with a slow onset, but a prolonged effect (frovatriptan), may overcome the need to treat all attacks at the earliest opportunity. Indeed, there is controversy as to whether migraine patients should be advised to treat all attacks early with triptans [2, 19–23]. Rather, some authors suggest that patients should be free to take their medication as soon as they are sure they are developing a migraine headache, because this could reduce the risk of medication-overuse headaches and related adverse drug reactions [2, 20, 23]. In this regard a two-drug combination with synergistic activity, ensuring both quick and sustained pain free activity, should be regarded as a useful treatment option for migraineurs.

There are several additional interesting outcomes of our study which are worth discussion. The patients with late drug use had more frequently a history of migraine of moderate severity at baseline, whereas those with early drug intake reported more often severe attacks and associated phonophobia. Additionally, use of triptans was less often reported by early treatment patients. Both these findings may suggest that patients could be motivated to take the study drugs earlier because their attacks are usually more painful and because they are less used to a selective antimigraine drug, such as a triptan.

Patients taking the FroDex combination expressed a much better preference than those taking the monotherapy. Since the study had a double-blind design, such a finding further supports and strengthens the favorable efficacy results obtained with the combination.

The fact that significantly less patients in the late treatment group treated with FroDex37.5 needed rescue medication, as compared to patients taking frovatriptan alone, could be regarded as an additional beneficial treatment feature.

The post hoc nature of the analysis represents the main limitation of our work. Although we acknowledge that further, prospective randomized trials are needed we also wish to point out that this is the first study demonstrating that the use of a combination therapy based on a triptan and an NSAIDs with particular pharmacological features, may not necessarily imply the need for an early use of the drug after the attack to ensure a prompt and sustained pain free response.

In conclusion, our results suggest that the frovatriptan plus dexketoprofen combination is effective in treating acute migraine attacks irrespective of the time treatment is started after the onset of pain.
